# Multivessel versus IRA-only PCI in patients with NSTEMI and severe left ventricular systolic dysfunction

**DOI:** 10.1371/journal.pone.0258525

**Published:** 2021-10-13

**Authors:** Myunhee Lee, Dae-Won Kim, Mahn-Won Park, Kyusup Lee, Sung-Ho Her, Kiyuk Chang, Wook Sung Chung, Myung Ho Jeong, Seung-Woon Rha, Hyo-Soo Kim, Hyeon Cheol Gwon, In Whan Seong, Kyung Kuk Hwang, Shung Chull Chae, Kwon-Bae Kim, Young Jo Kim, Kwang Soo Cha, Seok Kyu Oh, Jei Keon Chae, Ji-Hoon Jung, on behalf of KAMIR-NIH registry investigators

**Affiliations:** 1 Division of Cardiology, Daejeon St. Mary’s Hospital, College of Medicine, The Catholic University of Korea, Daejeon, Republic of Korea; 2 Division of Cardiology, St. Vincent’s Hospital, College of Medicine, The Catholic University of Korea, Suwon, Republic of Korea; 3 Division of Cardiology, Seoul St. Mary’s Hospital, College of Medicine, The Catholic University of Korea, Seoul, Republic of Korea; 4 Chonnam National University Hospital, Gwangju, Republic of Korea; 5 Guro Hospital, Seoul, Republic of Korea; 6 Seoul National University Hospital, Seoul, Republic of Korea; 7 Sungkyunkwan University, Samsung Medical Center, Seoul, Republic of Korea; 8 Chungnam National University Hospital, Daejeon, Republic of Korea; 9 Chungbuk National University Hospital, Cheongju, Republic of Korea; 10 Kyungpook National University Hospital, Daegu, Republic of Korea; 11 Keimyung University Dongsan Medical Center, Daegu, Republic of Korea; 12 Yeungnam University Hospital, Daegu, Republic of Korea; 13 Pusan National University Hospital, Busan, Republic of Korea; 14 Wonkwang University Hospital, Iksan, Republic of Korea; 15 Chonbuk National University Hospital, Jeonju, Republic of Kore; 16 Korea Institute of Toxicology, Daejeon, Republic of Korea; Azienda Ospedaliero Universitaria Careggi, ITALY

## Abstract

**Background:**

A substantial number of patients presenting with non-ST-elevation myocardial infarction (NSTEMI) and multivessel disease (MVD) have severe left ventricular systolic dysfunction (LVSD) (left ventricular ejection fraction (LVEF) less than 35%). But data are lacking regarding optimal percutaneous coronary intervention (PCI) strategy for these patients. The aim of this study was to compare the long-term outcomes of IRA (infarct-related artery)-only and multivessel PCI in patients with NSTEMI and MVD complicated by severe LVSD.

**Methods:**

Among 13,104 patients enrolled in the PCI registry from November 2011 to December 2015, patients with NSTEMI and MVD with severe LVSD who underwent successful PCI were screened. The primary outcome was 3-year major adverse cardiovascular events (MACEs), defined as all-cause death, any myocardial infarction, stroke, and any revascularization.

**Results:**

Overall, 228 patients were treated with IRA-only PCI (n = 104) or MV-PCI (n = 124). The MACE risk was significantly lower in the MV-PCI group than in the IRA-only PCI group (35.5% vs. 54.8%; hazard ratio [HR] 0.561; 95% confidence interval [CI] 0.378–0.832; p = 0.04). This result was mainly driven by a significantly lower risk of all-cause death (23.4% vs. 41.4%; hazard ratio [HR] 0.503; 95% confidence interval [CI] 0.314–0.806; p = 0.004). The results were consistent after multivariate regression, propensity-score matching, and inverse probability weighting to adjust for baseline differences.

**Conclusions:**

Among patients with NSTEMI and MVD complicated with severe LVSD, multivessel PCI was associated with a significantly lower MACE risk. The findings may provide valuable information to physicians who are involved in decision-making for these patients.

## Introduction

Despite the advancement of preventive medical interventions, guideline-recommended early intervention strategies, and early detection methods such as high-sensitivity troponin, as well as widespread public awareness of acute myocardial infarction (AMI), the incidence of non-ST-elevation myocardial infarction (NSTEMI) has been increasing in the past decade, according to data from the United States [1], Europe [2] and South Korea [3]. Multivessel disease (MVD) is prevalent among acute myocardial infarction (AMI) patients, and it is identified in 40–50% of patients seen with STEMI [4, 5], and 40–70% of those seen with NSTEMI [6, 7]. The presence of MVD with severe left ventricular systolic dysfunction (LVSD) in AMI patients has long been associated with worse clinical outcomes [5, 8]. Recently, data from the British Cardiac Intervention Society (BCIS) PCI database [9] and Korea Acute Myocardial Infarction Registry [10, 11] showed significantly lower cumulative mortality rates in patients who underwent multivessel (MV)-PCI than in those who underwent infarct-related artery (IRA)-only PCI, demonstrating that LVSD is a strong predictor of all-cause mortality. Nevertheless, a minority of the population in both registries was affected by severe LVSD. Although the current guidelines recommend the MV-PCI strategy in NSTEMI patients with MVD, excluding those with cardiogenic shock [12, 13], the preferred revascularization strategy has not been defined, and the PCI strategy used in NSTEMI patients with severe LVSD (left ventricular ejection fraction (LVEF) ≤ 35%) was inconsistent, as the selection of the strategy depends on the operator due to the absence of robust clinical data. Therefore, we investigated the efficacy and safety of two reperfusion strategies, IRA-only PCI and MV-PCI, in patients with NSTEMI and MVD with severe LVSD in real-world practice.

## Methods

### Design and study population

The data were obtained from the Korea Acute Myocardial Infarction Registry–National Institutes of Health (KAMIR-NIH) database. The KAMIR-NIH is a prospective, multicenter, cohort study that included AMI patients from 20 tertiary university hospitals in Korea from November 2011 to December 2015. The detailed study protocols have been published previously [14]. Among the 13,104 patients, we analyzed patients with NSTEMI and MVD who presented with severe LVSD and underwent PCI. The definition of NSTEMI was based on the criteria for the fourth universal definition of MI [15]. MVD was defined as the presence of ≥ 70% diameter stenosis in two or more major epicardial arteries or ≥ 50% diameter stenosis in the left main coronary artery.

**Fig 1:**
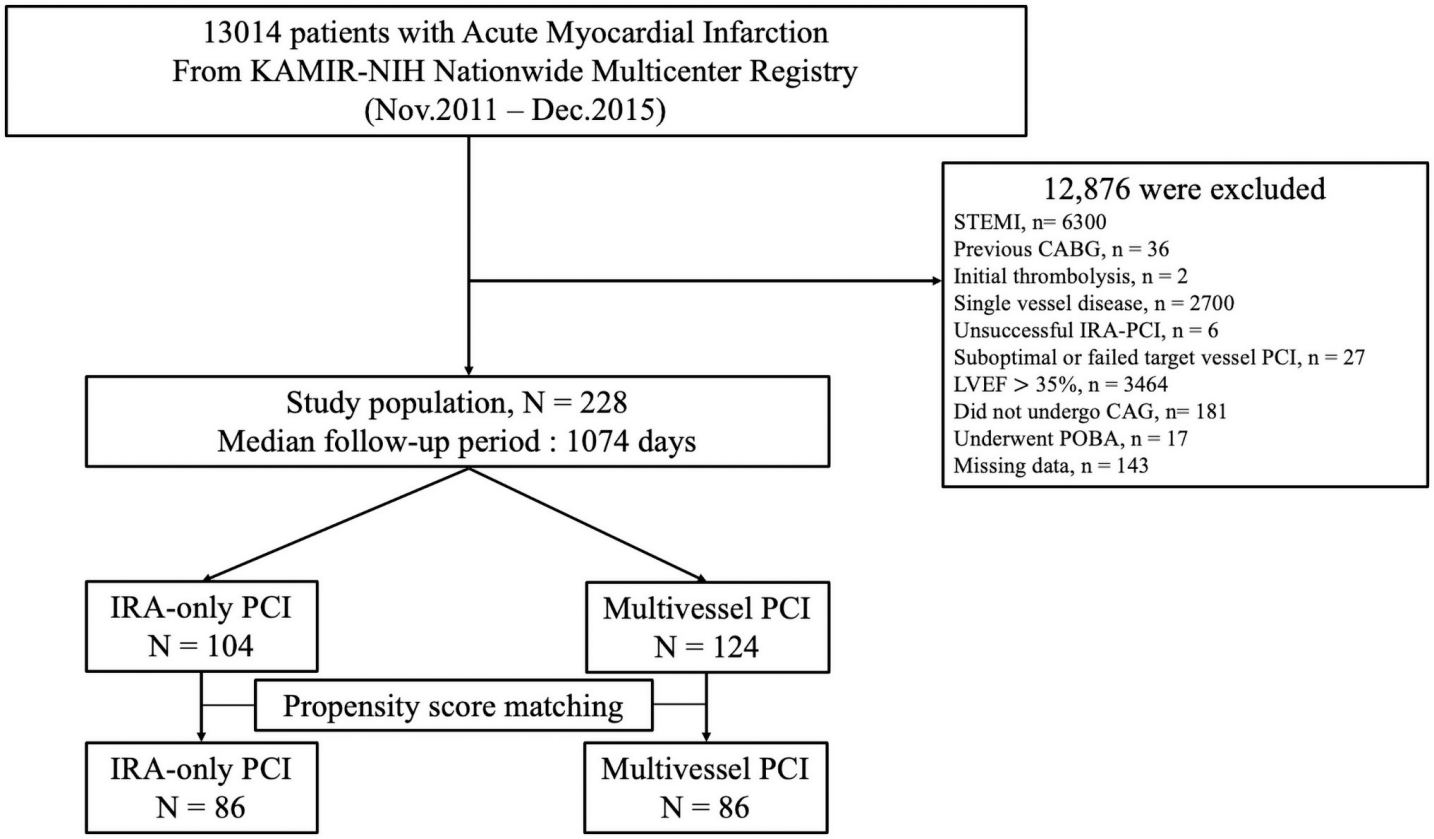
Study flowchart. The study population selection and the numbers of patients included and excluded in the present study are shown. STEMI, ST-segment elevation myocardial infarction; CABG, coronary artery bypass graft; IRA, infarct-related artery; PCI, percutaneous coronary intervention; LVEF, left ventricular ejection fraction; CAG, coronary angiography; POBA, plain old balloon angioplasty.

Global LV systolic function was qualitatively measured based on a 2-dimensional echocardiogram as the LVEF. Severe LVSD was defined as LVEF ≤ 35% based on inclusion criteria of STICH (Surgical Treatment for Ischemic Heart Failure) trial [16] and current recommendations for revascularization (2013 ACC/AHA guideline for heart failure [17] and 2018 ESC/EACTS Guidelines on myocardial revascularization [18]). PCI was considered successful if the final residual stenosis was <30% with thrombolysis in myocardial infarction (TIMI) grade 3 flow. To compare the effect of two PCI strategies (MV-PCI or IRA-only PCI) in patients with NSTEMI and MVD with severe LVSD, we only included who successfully underwent PCI during initial hospitalization. We excluded 216 patients (33 patients who had unsuccessful or failed PCI, 2 patients who received thrombolysis, and 181 patients who did not undergo CAG) who were considered inappropriate candidates for CAG (and PCI). Other exclusion criteria were STEMI, patients who had a history of coronary artery bypass grafting (CABG), had single-vessel disease, or presented with a LVEF > 35% at presentation. Finally, 228 patients were included for analysis and stratified according to revascularization strategy (i.e., IRA-only PCI or MV-PCI) (Fig 1). MV-PCI was defined as IRA PCI followed by either non-IRA PCI at the time of index PCI or staged non-IRA PCI during initial hospitalization. After PCI, dual antiplatelet therapy was prescribed and maintained for at least 1 year unless there was any reason for its discontinuation. Patients were managed according to current NSTEMI guidelines [18, 19]. Cardiogenic shock was defined as systolic blood pressure <90 mm Hg for >30 min or the need for inotropes to maintain systolic blood pressure >90 mm Hg; signs of pulmonary congestion; and signs of impaired organ perfusion with at least 1 of the following: cool extremities, decreased urine output < 30ml/h, increased lactic acid level (> 2.0 mmol/l), or altered mental status. The complexity of coronary lesions was scored based on the definitions of the American College of Cardiology (ACC)/American Heart Association (AHA). Complex coronary anatomy (or complex lesion) was defined as ACC/AHA type B2 or C lesions [20]. CKD (chronic kidney disease) was defined as GFR ≤ 59 mL/min/1.73m^2^. The eGFR (estimated glomerular-filtration rate) was calculated using the Cockroft-Gault Formula. The choice of PCI strategy, prescribed medications, approach site, use of intravascular imaging, procedural application related to PCI such as stents (type, diameter, length, and the number of stents), aspiration thrombectomy, or hemodynamic support devices were left to the discretion of operator. Clinical events that occurred during index hospitalization and within the 3-year follow-up were analyzed. This study was approved by the institutional review board (IRB) of each participating institution (IRB of the Catholic University of Korea, Daejeon, St. Mary’s hospital, IRB of the Catholic University of Korea, Suwon, St. Vincent’s Hospital, IRB of the Catholic University of Korea, Seoul, St. Mary’s hospital, IRB of Chonnam National University, IRB of Korea University Guro IVD Support Center, IRB of Seoul National University Hospital Biomedical Research Institute, IRB of Samsung Medical Center Clinical Trial Center, IRB of Chungnam National University Hospital, IRB of Chungbuk National University Hospital, IRB of Kyungpook National University Hospital, Clinical Trial Center of Keimyung University Dongsan Medical Center, Clinical Trial Center for Medical Devices of Yeungnam University Hospital, Clinical Trial Center of Pusan National University Hospital, IRB of Wonkwang University Hospital and IRB of Chonbuk National University Hospital) and was conducted according to the Declaration of Helsinki. Written informed consent was obtained from all participants.

### Outcomes

The primary outcome was major adverse cardiac events (MACEs) at 3 years (a composite of all-cause death, any myocardial infarction (MI), stroke, or any repeat revascularization). The secondary outcomes were the individual components of cardiac death, non-IRA repeat revascularization, or any rehospitalization at 3 years. In-hospital outcomes were defined as cardiogenic shock, recurrent MI, newly developed heart failure, stent thrombosis, and cerebrovascular events (a composite of cerebral infarction and intracranial hemorrhage). In-hospital complications included TIMI major and minor bleeding, acute kidney injury (AKI), ventricular tachycardia (VT), or ventricular fibrillation (VF). All deaths were considered cardiac deaths unless a definite noncardiac cause was identified. Recurrent MI was defined as the recurrence of angina accompanied by changes in electrocardiogram or an increase in cardiac enzyme levels. Repeat revascularization was documented as clinically driven revascularization that happened after discharge from the index hospitalization. AKI was defined as a reduction in kidney function (absolute increase in serum creatinine of ≥ 0.3 mg/dl, a percentage increase in serum creatinine ≥ 50% or a decrease in urine output ≤ 0.5 ml/kg/h for more than 6 h) less than 48 h after PCI. All clinical outcomes were defined according to the Academic Research Consortium guidelines.

### Statistical analysis

Continuous variables are expressed as the means ± standard deviations when normally distributed or as the medians (interquartile ranges) if nonnormally distributed. Categorical data are presented as absolute values and percentages. Differences in categorical variables between groups were analyzed with the chi-square test or Fisher’s exact test as appropriate, and differences in continuous variables between groups were analyzed with 2-tailed Student`s t-tests or the Mann-Whitney U tests. Stratified by the revascularization strategy, the primary outcome was estimated at 3 years. The data are presented in tables. Kaplan-Meier curves were constructed. The log-rank test was performed to compare the incidences of the outcomes between the groups stratified by revascularization strategy. Based on the variables that were significant (P < 0.05), propensity score-matching analysis was performed to reduce bias due to confounding variables. Baseline demographic, clinical, and angiographic characteristics were compared within the propensity score-matched group. The propensity score was computed by nonparsimonious multiple logistic regression analysis (c-statistics = 0.668). Matching was performed using 1:1 nearest- neighbor matching from an initial 7 to 1 digit. For inverse probability of treatment weighting (IPTW) adjustment, the inverse of the propensity score was assessed by calculating the standardized mean differences in the covariate used to generate the propensity score. We confirmed the model reliability with the goodness-of-fit test (p = 0.771). In the matched cohort, paired comparisons were performed using McNemar’s test for binary variables and a paired t-test for continuous variables. All analyses were two-tailed, and clinical significance was defined as p < 0.05. All statistical analyses were performed using Statistical Analysis Software (SAS, version 9.4, SAS Institute, Cary, NC, USA).

## Results

Of the 13,104 patients enrolled in the KAMIR-NIH registry, 6,192 patients were diagnosed with NSTEMI. Among them, 3486 (56.3%) patients had MV disease. Among the 6,192 NSTEMI patients, 437 patients were diagnosed with NSTEMI with severe LVSD. Among them, 338 (77.3%) patients had MV disease (S1 Fig). Patients were excluded from the analysis if they met one of the exclusion criteria (Fig 1). Finally, 228 patients were selected for inclusion in the study. Among these patients, 104 (45.6%) underwent IRA-only PCI, and 124 (54.4%) underwent MV-PCI. The median follow-up duration was 1074 days (Fig 1).

### Baseline characteristics

The baseline clinical, lesional and procedural characteristics are described in **Tables** 1 **and** 2 (baseline characteristics of the propensity score-matched cohort are listed in S1 **and** S2 **Tables**). The study population had a mean age of 69.5 years, and 68.4% were men; 52.1% of the patients had diabetes, 61.8% had hypertension, 26.8% were current smokers, and 53.0% had chronic kidney disease (CKD). The majority of PCI procedures were performed through the radial route, accounting for 64.9% of the procedures. Most of the patients who underwent PCI received second-generation drug-eluting stent (DES)s. Of these patients, 104 (45.6%) underwent IRA-only PCI, and 124 (54.4%) underwent MV-PCI. Overall, there was no significant difference in baseline characteristics between the two groups, with the exception that those undergoing IRA-only PCI were older, were less likely to have received potent P2Y12 inhibitors and had a lower left main (LM) disease incidence in the overall lesion profile. The median time interval between the diagnosis and CAG was 2.2 ± 4.8 days without a significant difference between the two groups (1.9 ± 3.1 days in the MV-PCI group vs. 2.6 ± 6.2 days in the IRA-only PCI group, p = 0.951). There were also no differences in the time from symptom onset to revascularization between the groups.

**Table 1:** Baseline demographic, clinical and laboratory characteristics in the study population stratified by revascularization strategy.

		Crude population		
Characteristic	Total population	IRA-Only PCI	Multivessel PCI	p-value
	(n = 228)	(n = 104)	(n = 124)	
**Demographic**				
** Age (years)**	69.5±11.1	71.1±10.7	68.1±11.2	0.045
** Age > 70 years**	129(56.6)	65(62.5)	64(51.6)	0.099
** Male**	156(68.4)	74(71.2)	82(66.1)	0.416
** BMI (kg/m** ^**2**^ **)**	22.8±3.6	22.9±4.2	22.7±3.1	0.744
**Initial presentation**				
** SBP (mmHg)**	126.6±34.0	126.5±34.5	126.7±33.7	0.965
** DBP (mmHg)**	76.4±20.8	76.2±21.2	76.6±20.5	0.884
** Heart rate (frequency/min)**	95.3±25.0	95.9±23.8	94.8±26.1	0.744
** Killip classification (%)**	97(42.5)	43(41.4)	54(43.6)	0.738
** **III, IV	126.6±34.0	126.5±34.5	126.7±33.7	0.965
** Symptom onset-to-arrival time, days**	1.6±3.1	1.3±1.8	1.8±3.9	0.386
** Arrival-to-angiography time, days**	2.2±4.8	2.6±6.2	1.9±3.1	0.951
**Clinical risk factors**				
** Diabetes (%)**	121(53.1)	57(54.8)	64(51.6)	0.630
** Hypertension (%)**	141(61.8)	62(59.6)	79(63.7)	0.526
** Dyslipidemia (%)**	22(9.6)	8(7.7)	14(11.3)	0.359
** Previous MI (%)**	32(14.0)	16(15.4)	16(12.9)	0.591
** Prior CVA (%)**	32(14.0)	16(15.4)	16(12.9)	0.591
** CKD (%)**	121(53.1)	54(51.9)	67(54.0)	0.751
** eGFR (mL/min/1.72m** ^**2**^ **)**	34.9±16.9	34.4±14.6	35.3±18.6	0.780
** Stage 3**	78(34.2)	35(33.7)	43(34.7)	
** Stage 4**	18(7.9)	12(11.5)	6(4.8)	
** Stage 5**	25(11.0)	7(6.7)	18(14.5)	
** Current smoking (%)**	61(26.8)	22(21.2)	39(31.5)	0.080
**Laboratory findings**				
** Hb (g/dL)**	12.6±2.2	12.4±2.2	12.7±2.3	0.352
** Creatinine (mg/dL)**	1.9±1.9	1.8±1.7	1.9±2.0	0.602
** eGFR (mL/min/1.72m** ^**2**^ **)**	60.4±34.1	59.7±32.7	61.0±35.4	0.772
** HbA1c (%)**	7.0±1.7	6.8±1.5	7.1±1.8	0.219
** CK-MB (ng/mL)**	69.1±112.3	66.5±119.4	71.3±106.5	0.349
** Troponin I (ng/mL)**	36.3±77.9	27.9±63.0	43.0±88.0	0.187
** LDL cholesterol (mg/dL)**	102.3±39.5	95.7±34.1	107.5±42.7	0.036
** HDL cholesterol (mg/dL)**	40.8±11.7	42.0±11.7	39.8±11.7	0.182
** hsCRP (mg/L)**	3.1±4.5	2.9±4.5	3.3±4.6	0.218
** NT-proBNP (pg/mL)**	10,109.9±10,809.1	10,273.6±11,251.5	9,938.2±10,391.4	0.900
** LVEF (%)**	27.9±5.0	27.8±4.7	28.0±5.3	0.819
**Medications at discharge**				
** Aspirin (%)**	221(96.9)	100(96.2)	121(97.6)	0.705
** Clopidogrel (%)**	183(80.3)	91(87.5)	92(74.2)	0.012
** Prasugrel (%)**	15(6.6)	3(2.9)	12(9.7)	0.039
** Ticagrelor (%)**	23(10.1)	7(6.7)	16(12.9)	0.123
** Potent P2Y12 inhibitors (%)**	38(16.7)	10(9.6)	28(22.6)	0.009
** ACE inhibitor/ARB (%)**	170(74.6)	75(72.1)	95(76.6)	0.437
** ß-blocker (%)**	179(78.5)	78(75.0)	101(81.5)	0.238
** Statin (%)**	190(83.3)	83(79.8)	107(86.3)	0.191
** Oral anticoagulant (%)**	22(9.6)	9(8.7)	13(10.5)	0.641

Data are presented as mean ± SD, median (interquartile range), and number (percentage) as appropriate.

Abbreviations: IRA, infarct related artery; BMI, body mass index; SBP, systolic blood pressure; DBP, diastolic blood pressure; MI, myocardial infarction; CVA, cerebrovascular accident; CKD, chronic kidney disease; Hb, hemoglobin; eGFR, estimated glomerular filtration rate; HbA1c, glycated hemoglobin A1c; LDL, low-density lipoprotein; HDL, high-density lipoprotein; hsCRP, high-sensitivity C-reactive protein; NT-proBNP, N-terminal prohormone of brain natriuretic peptide; LVEF, left ventricular ejection fraction; ACE inhibitor, angiotensin-converting enzyme inhibitor; ARB, angiotensin receptor blocker.

**Table 2:** Baseline lesional and procedural characteristics in the study population stratified by revascularization strategy.

Crude Population				
Characteristic	Total	IRA-Only PCI	Multivessel PCI	p-value
	(n = 228)	(n = 104)	(n = 124)	
**Culprit vessel (%)**				0.188
** **LAD	93(40.8)	50(48.1)	43(34.7)	
** **LCX	42(18.4)	18(17.3)	24(19.4)	
** **RCA	69(30.3)	28(26.9)	41(33.1)	
** **LMCA	24(10.5)	8(7.7)	16(12.9)	
**Lesion classification (%)**				
** B2/C**	206(90.4)	92(88.5)	114(91.9)	0.376
** Small vessel**	110(48.2)	47(45.2)	63(50.8)	0.398
** Long lesion**	118(51.8)	48(46.2)	70(56.5)	0.121
**Overall lesion profile (%)**				
** Left main disease**	41(18.0)	13(12.5)	28(22.6)	0.048
** Three-vessel disease**	105(46.1)	50(48.1)	55(44.4)	0.574
**Pre TIMI flow of culprit vessel (%)**				0.328
** **0	58(25.4)	22(21.2)	36(29.0)	
** **I	34(14.9)	18(17.3)	16(12.9)	
** **II, III	136(59.6)	64(61.5)	72(58.1)	
**Post TIMI flow of culprit vessel (%)**				
** **II, III	228(100.0)	104(100.0)	124(100.0)	
**IVUS during PCI (%)**	48(21.1)	17(16.4)	31(25.0)	0.110
**OCT during PCI (%)**	2(0.9)	2(1.9)	0(0.0)	0.207
**IRA treatment (%)**				0.181
** **Bare-metal stent	8(3.5)	5(4.8)	3(2.4)	
** **First-generation DES	2(0.9)	2(1.9)	0(0.0)	
** **Second-generation DES	218(95.6)	97(93.3)	121(97.6)	
**Total number of implanted stents**	2.0±1.0	1.3±0.6	2.6±1.0	<0.001
**Timing of non-IRA PCI**				
** **Single-staged PCI during index procedure	-	-	84(67.7)	-
** **Multi-staged PCI during index hospitalization	-	-	40(32.3)	-
**Complete revascularization**	-	-	75(60.5)	-
**Hemodynamic support**				
** **Intra-aortic balloon pump	15(6.6)	5(4.8)	10(8.1)	0.323
** **Extracorporeal membrane oxygenation	6(2.6)	4(3.9)	2(1.6)	0.416
**Approach site**				
** **Transfemoral approach	80(35.1)	36(34.6)	44(35.5)	0.891
**Glycoprotein IIb/IIIa inhibitor**	22(9.6)	13(12.5)	9(7.3)	0.182
**Thrombus aspiration**	20(8.8)	10(9.6)	10(8.1)	0.680

Data are presented as number (percentage) where appropriate.

Abbreviations: LAD, left anterior descending artery; LCX, left circumflex artery; RCA, right coronary artery.

LMCA, left main coronary artery, lesion based on American College of Cardiology/American Heart Association. lesion classification; TIMI, thrombolysis in myocardial infarction; IVUS, intravascular ultrasound; OCT, optical coherence tomography; DES, drug eluting stent; PCI, percutaneous coronary intervention; GpIIb-IIIa inhibitor, glycoprotein IIb/IIIa inhibitor.

### In-hospital clinical outcomes and complications

There were no significant differences between the two groups in in-hospital outcomes. There was a higher proportion of patients with cardiogenic shock in the IRA-only PCI group but a lower proportion with newly developed heart failure after PCI; however, neither difference was statistically significant. No significant differences were observed in in-hospital complications, such as TIMI major or minor bleeding, AKI, or life-threatening arrhythmia (e.g., VT, VF) (Table 3, **upper panel**). There were no differences in in-hospital outcomes or in-hospital complications between the two groups in the propensity score-matched cohort (Table 3, **lower panel**).

**Table 3:** In-hospital clinical outcomes and complications stratified by revascularization strategy in the overall and propensity score-matched populations.

**Crude population**								
	**IRA-Only PCI**	**Multivessel PCI**	**p-value**	**Log rank p-value**	**HR**	**95.0% CI**		**p-value**
	**No. (%)**	**No. (%)**						
	**(n = 104)**	**(n = 124)**				**Lower**	**Upper**	
**Cardiogenic shock**	14(13.5)	16(12.9)	0.901	0.903	0.958	0.468	1.963	0.907
**Newly developed heart failure**	20(19.2)	30(24.2)	0.367	0.369	1.263	0.717	2.225	0.418
**Stent thrombosis**	1(1.0)	1(0.8)	>0.999	0.901	0.839	0.052	13.409	0.901
**Recurrent myocardial infarction**	1(1.0)	1(0.8)	>0.999	0.901	0.839	0.052	13.409	0.901
**Cerebral infarction**	0(0.0)	2(1.6)	0.502	0.201	-	-	-	-
**ICH**	3(2.9)	4(3.2)	>0.999	0.906	1.094	0.245	4.889	0.906
**Emergent CABG**	2(1.9)	0(0.0)	0.207	0.114	-	-	-	-
**TIMI major bleeding**	3(2.9)	5(4.0)	0.730	0.666	1.368	0.327	5.726	0.668
**TIMI minor bleeding**	4(3.9)	7(5.7)	0.528	0.551	1.449	0.424	4.949	0.554
**Acute kidney injury**	3(2.9)	6(4.8)	0.515	0.466	1.662	0.416	6.643	0.473
**VT/VF**	9(8.7)	12(9.7)	0.790	0.804	1.113	0.469	2.641	0.809
**Propensity Score Matching**								
	**IRA-Only PCI No. (%)**	**Multivessel PCI No. (%)**	**p-value**	**Log rank P-value**	**HR**	**95.0% CI**		**p-value**
	**(n = 86)**	**(n = 86)**				**Lower**	**Upper**	
**Cardiogenic shock**	12(14.0)	9(10.5)	0.648	0.486	0.747	0.315	1.773	0.509
**Newly developed heart failure**	16(18.6)	24(27.9)	0.201	0.151	1.510	0.802	2.843	0.201
**Stent thrombosis**	1(1.2)	0(0.0)	>0.999	0.317	-	-	-	-
**Recurrent myocardial infarction**	1(1.2)	0(0.0)	>0.999	0.317	-	-	-	-
**Cerebral infarction**	0(0.0)	1(1.2)	>0.999	0.326	-	-	-	-
**ICH**	2(2.3)	3(3.5)	>0.999	0.669	1.474	0.246	8.824	0.671
**Emergent CABG**	2(2.3)	0(0.0)	0.500	0.146	-	-	-	-
**TIMI major bleeding**	2(2.3)	3(3.5)	>0.999	0.669	1.474	0.246	8.824	0.671
**TIMI minor bleeding**	3(3.5)	5(5.8)	0.727	0.505	1.617	0.386	6.765	0.511
**Acute kidney injury**	2(2.3)	2(2.3)	>0.999	0.993	0.991	0.140	7.034	0.993
**VT/VF**	7(8.1)	5(5.8)	0.754	0.539	0.704	0.223	2.218	0.549

Data are presented as n (%).

Abbreviations: PCI, percutaneous coronary intervention; CI, confidence interval; HR, hazard ratio.

ICH,intracranial hemorrhage; CABG, coronary artery bypass grafting; TIMI, thrombolysis in myocardial infarction; VT, ventricular tachycardia; VF, ventricular fibrillation.

### Long-term clinical outcomes

**Fig 2:**
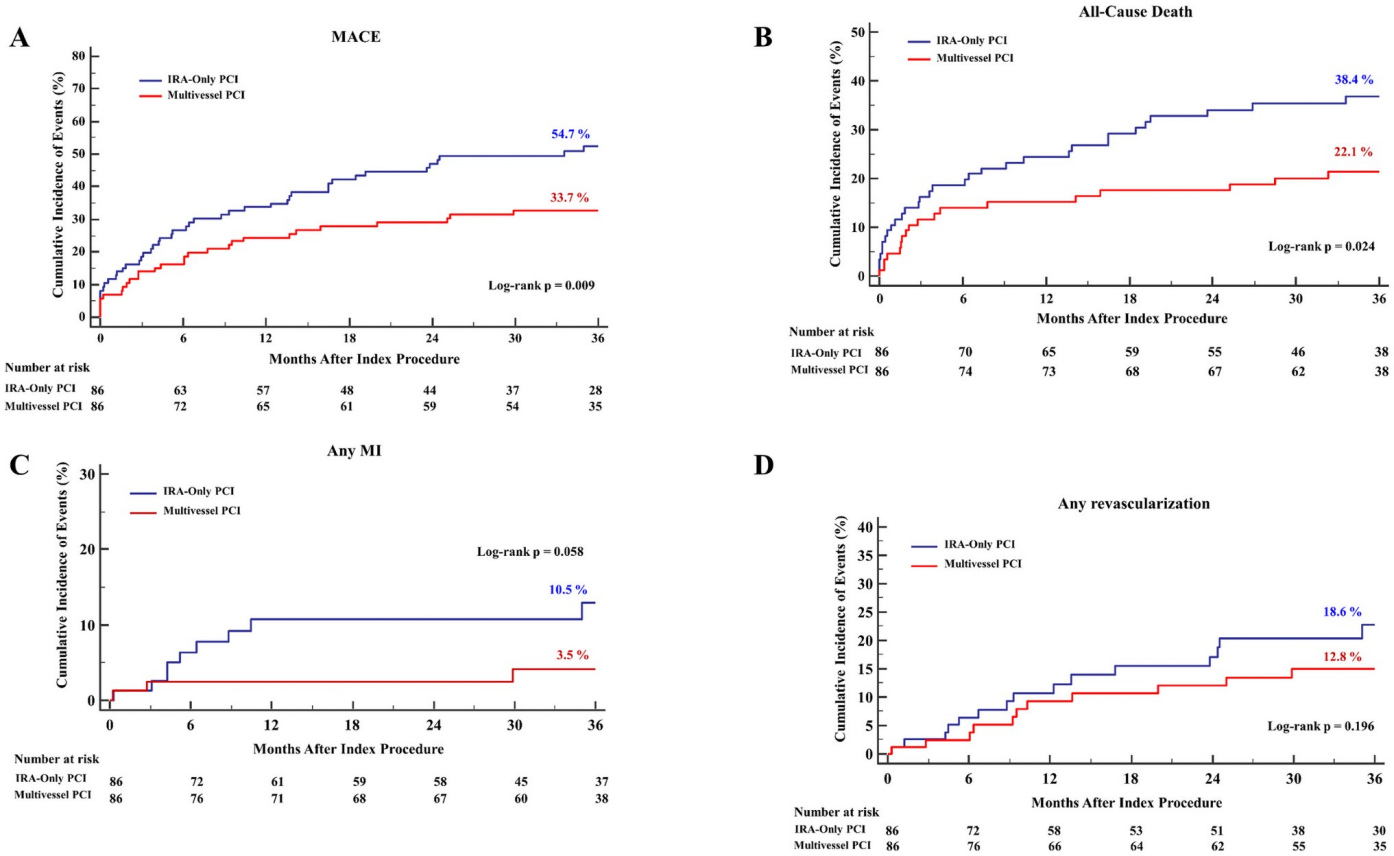
Three-year cumulative incidence of primary outcomes and individual components in propensity-matched population. Kaplan-Meier curves for (A) MACEs, (B) all-cause mortality, (C) any MI, and (D) any revascularization according to the PCI strategy are shown. MACEs, major adverse cardiac events; MI, myocardial infarction; PCI, percutaneous coronary intervention.

The 3-year clinical outcomes stratified by revascularization strategy were shown in Table 4. The risk of MACEs was significantly lower in the MV-PCI group than in the IRA-only PCI group (35.5% vs. 54.8%; HR (95% CI): 0.56 (0.38–0.83); p = 0.004) and was mainly driven by a significantly lower risk of all-cause mortality in the MV-PCI group. The risk of cardiac death was also significantly lower in the MV-PCI group than in the IRA-only PCI group (14.5% vs. 30.8%; HR (95% CI): 0.43 (0.24–0.76); p = 0.004). Sensitivity analysis performed with multivariate Cox regression, propensity-score matching, and IPTW adjustment consistently showed a significantly lower risk of MACEs, all-cause mortality, and cardiac death in the MV-PCI group than in the IRA-only PCI group (Table 4). The 3 years clinical outcomes of two reperfusion strategy in propensity-score matched population are depicted in Fig 2. The MV-PCI groups showed a significantly lower risk of MACEs and all-cause death compared with the IRA-only groups. The risk of any MI and any revascularization also showed a similar trend: patients treated by the MV-PCI experienced fewer events than those treated by the IRA-only PCI throughout the study period.

**Table 4:** Three-year primary and secondary clinical outcomes stratified by revascularization strategy.

	IRA-Only PCI	Multivessel PCI	Unadjusted		Multivariable Adjusted		Propensity Score Matched		IPTW-Adjusted	
	(n = 104)	(n = 124)	HR (95% CI)	P value	HR (95% CI)	P value	HR (95% CI)	P value	HR (95% CI)	P value
**Primary outcomes**										
**MACE**	57(54.8)	44(35.5)	0.56(0.38–0.83)	0.004	0.52(0.34–0.79)	0.002	0.54(0.34–0.87)	0.010	0.60(0.45–0.80)	0.001
** All-cause death**	43(41.4)	29(23.4)	0.50(0.31–0.81)	0.004	0.49(0.30–0.81)	0.005	0.53(0.30–0.93)	0.027	0.54(0.39–0.76)	<0.001
** Any MI**	9(8.7)	9(7.3)	0.76(0.30–1.91)	0.553	0.47(0.17–1.34)	0.159	0.30(0.08–1.12)	0.074	0.74(0.37–1.49)	0.398
** Stroke**	1(1.0)	1(0.8)	0.81(0.05–12.92)	0.880	1.48(0.08–26.66)	0.790	0.97(0.06–15.56)	0.985	1.31(0.21–8.13)	0.774
** Any revascularization**	17(16.4)	17(13.7)	0.73(0.37–1.43)	0.355	0.71(0.35–1.43)	0.336	0.61(0.28–1.31)	0.201	0.81(0.49–1.34)	0.411
**Secondary outcomes**										
** Cardiac death**	32(30.8)	18(14.5)	0.43(0.24–0.76)	0.004	0.38(0.21–0.70)	0.002	0.29(0.13–0.64)	0.002	0.40(0.26–0.61)	<0.001
** Non-IRA revascularization**	5(4.8)	7(5.7)	1.01(0.32–3.18)	0.986	1.34(0.41–4.40)	0.631	1.13(0.08–1.12)	0.861	1.32(0.57–3.05)	0.513
** Any rehospitalization**	13(12.5)	21(16.9)	1.30(0.65–2.60)	0.459	1.24(0.60–2.57)	0.569	1.06(0.43–2.61)	0.899	1.48(0.89–2.47)	0.131

Data are presented as n (%).

Abbreviations: IPTW, inverse probability of treatment weighting; CI, confidence interval; HR, hazard ratio; MACE, major adverse cardiac event; MI, myocardial infarction; IRA, infarct-related artery.

### Independent predictors of MACEs

A multivariate Cox proportional hazard model identified independent predictors of MACEs (S3 Table). In the multivariable logistic regression model, MV-PCI, statin use, and troponin I were independent predictors of MACEs. The MV-PCI was independently associated with a lower MACE risk (HR (95% CI): 0.576 (0.335–0.990); p = 0.046) than the IRA-only PCI at 3 years.

### Landmark analysis

**Fig 3:**
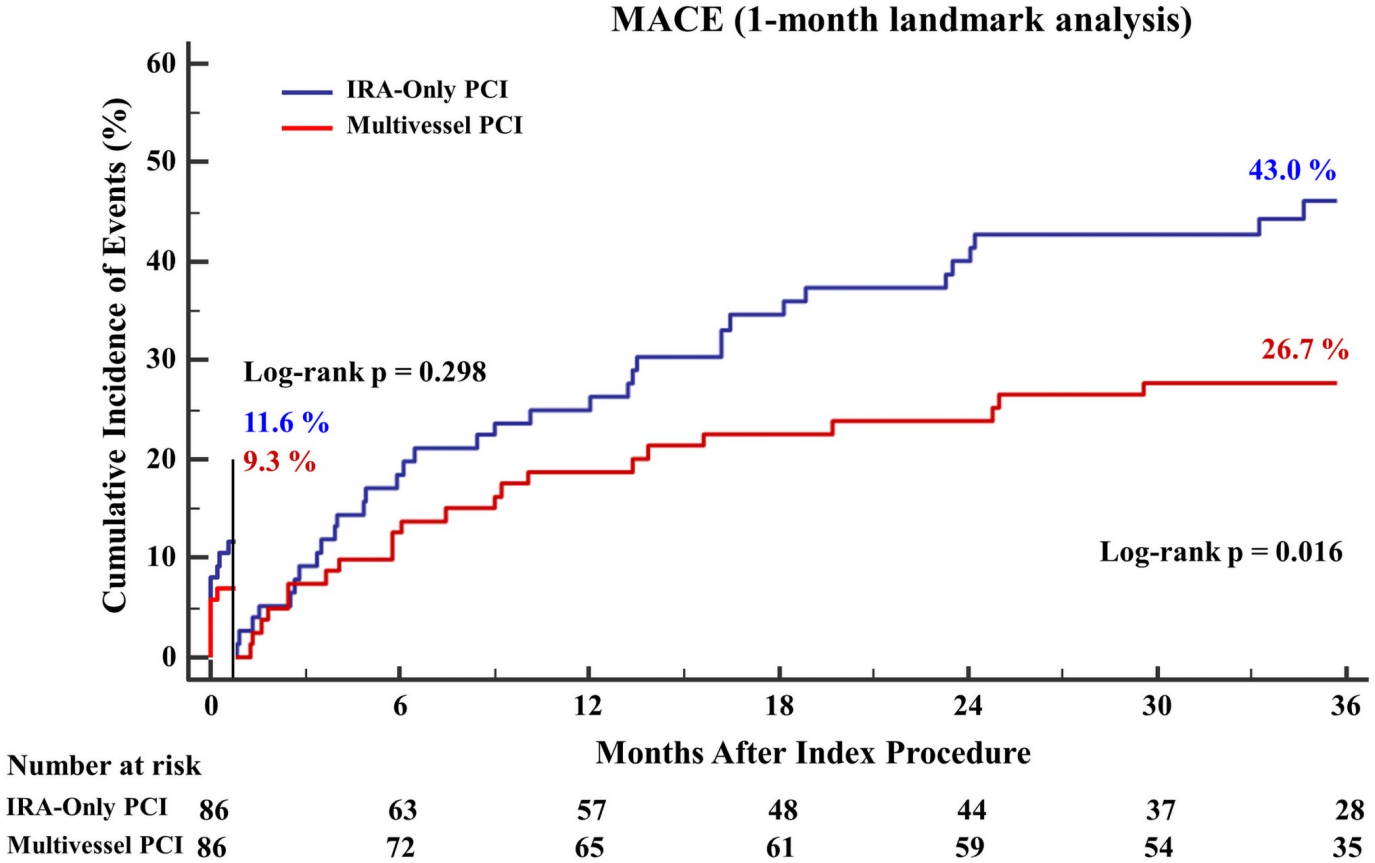
Three-year cumulative incidence of MACEs (1-month landmark analysis) in a propensity score-matched population. Kaplan-Meier curves with the 1-month landmark analysis of MACEs stratified by the PCI strategy are shown.

The Kaplan-Meier curves for 1-month and 3-year MACEs for the propensity score-matched cohort are shown in Fig 3. The landmark analysis revealed that the difference in MACEs based on the revascularization strategy mostly occurred after 1 month. The 1-month landmark analysis showed that there was no significant difference in the risk of MACEs between the MV-PCI group and the IRA-only PCI group (11.6% vs. 7.0%; HR (95% CI): 0.60(0.22–1.64); p = 0.424). However, beyond 1 month, patients in the IRA-only PCI group consistently had a higher risk of MACEs than patients in the MV-PCI group (43.0% vs. 26.7%; HR (95% CI): 0.43(0.32–0.90); p = 0.039).

### Subgroup analysis

**Fig 4:**
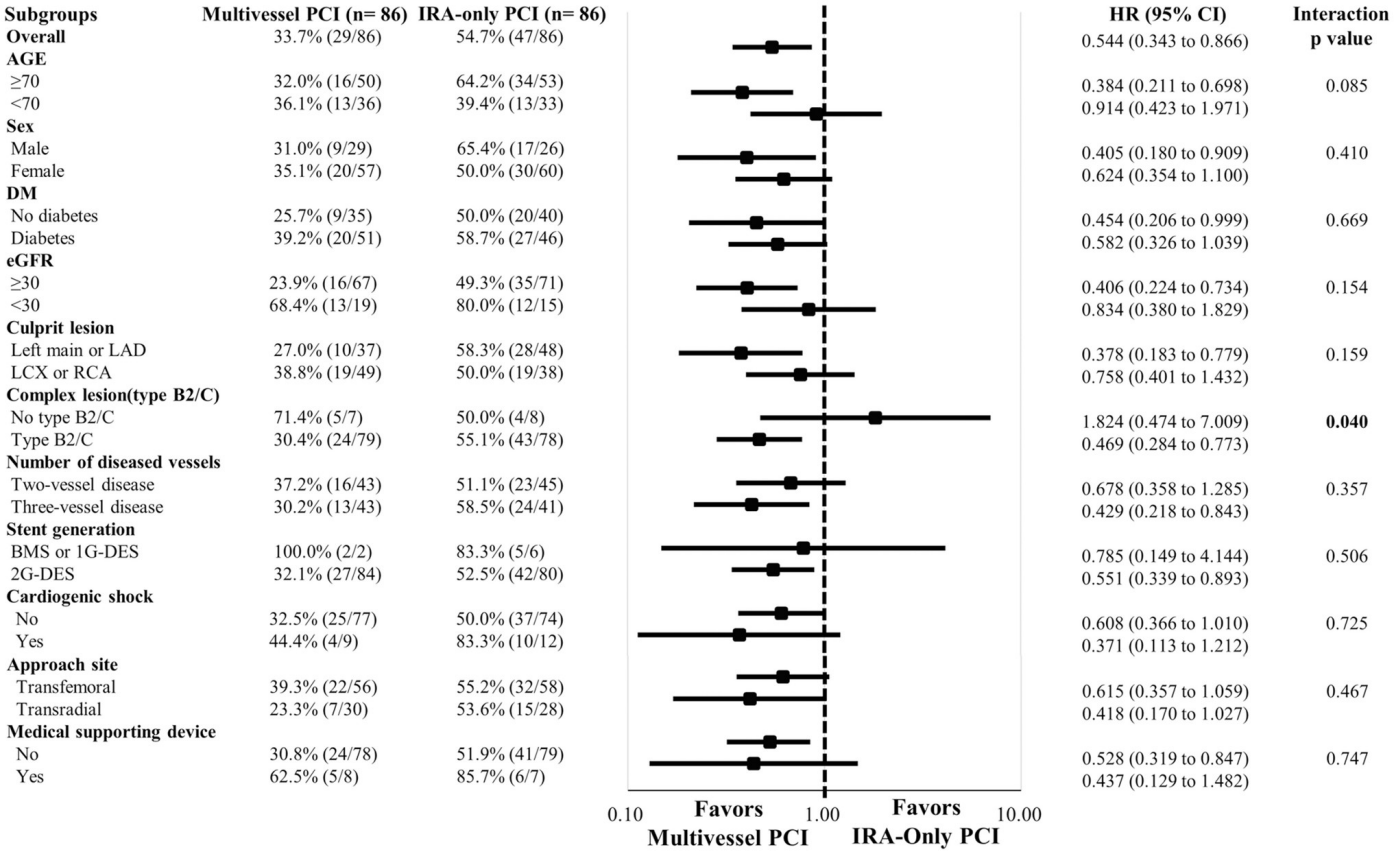
Exploratory subgroup analysis of MACEs. Prespecified subgroup analysis revealed consistent results across all the subgroups except for with regard to the complexity of the lesion profile. The beneficial effect of MV-PCI on the risk of MACEs was observed in only patients with complex coronary anatomy (Type B2/C lesion). HR, hazard ratio; CI, confidence interval; DM, diabetes mellitus; GFR, glomerular filtration rate; LAD, left anterior descending artery; LCX, left circumflex artery; RCA, right coronary artery; BMS, bare-metal stent; DES, drug-eluting stent.

Prespecified subgroup analyses (Fig 4) revealed that the MV-PCI group consistently had a significantly lower risk of MACEs than the IRA-only PCI group across almost all subgroups without significant interactions except with the presence of a complex lesion (type B2/C lesion), for which a trend toward a revascularization strategy-by-subgroup interaction was found. Among patients with complex lesion profiles, the MV-PCI group had a significantly lower risk of MACEs than the IRA-only PCI group (HR (95% CI): 0.469(0.284–0.773)). However, among patients without complex lesion profiles, a more favorable effect of the MV-PCI strategy than the IRA-only strategy was not observed (HR (95% CI): 1.824(0.474–7.001)) (P = 0.04 for interaction), implying that IRA-only PCI could be better in patients with relatively simple coronary lesions. Notably, the beneficial effect of the MV-PCI strategy was observed irrespective of age, sex, CKD severity, diabetes status, target vessel, number of diseased vessels, approach site (transfemoral or transradial), the presence of cardiogenic shock, or the use of mechanical support devices.

## Discussion

In this study, we evaluate the 3-year clinical outcomes between MV-PCI and IRA-only PCI in patients with NSTEMI and MVD accompanied by severe LVSD. The principal findings of this study can be summarized as follows. First, MVD is prevalent in patients with NSTEMI and severe LVSD and MV-PCI is associated with a lower long-term risk of MACEs than the IRA-only PCI. This result was driven by a significantly lower risk of all-cause mortality in the MV-PCI group, mainly due to a lower incidence of cardiac death in the MV-PCI group. These findings were consistently observed in multiple sensitivity analyses with adjustment for baseline differences. Second, MV-PCI also yielded comparable in-hospital clinical outcomes and complications to the IRA-only PCI strategy. Third, the beneficial effect of the MV-PCI strategy was observed 1 month after the index hospitalization and persisted for 3 years. Fourth, a significantly lower risk of MACEs in the MV-PCI group than in the IRA-only PCI group was consistently observed across various subgroups without significant interactions.

### Potential benefits of the MV-PCI strategy for patients with NSTEMI and severe LVSD

The 2014 American College of Cardiology Foundation/American Heart Association guidelines gives a class IIb recommendation for PCI in NSTEMI patients but no PCI recommendations for patients with severe LVSD [13]. Additionally, the latest 2020 ESC Acute Coronary Syndromes (ACS) in Patients Presenting without Persistent ST-Segment Elevation (Management of) Guidelines recommend complete revascularization in non-ST-elevation acute coronary syndrome (NSTE-ACS) patients without cardiogenic shock and with MV coronary artery disease (CAD) but offer no recommendations for patients with severe LVSD [12]. Until now, there has been a lack of randomized trials and recommendations regarding the optimal revascularization strategies in NSTEMI patients with severe LVSD. The identification of the IRA can be more challenging in patients with NSTEMI than in those with STEMI because the former patients are more likely to present with either MV CAD or insignificant CAD [21]. In patients with NSTEMI and LVSD, the use of the IRA-only PCI strategy could mean that culprit lesions are left untreated more often than when MV-PCI strategy is used. Revascularization provides more substantial benefits with regard to improving survival and quality of life, particularly in patients with more extensive MVD and the most severe phase of LVSD [22], regardless of whether viable myocardium is present [23, 24]. Additionally, revascularization may provide long-term benefits even without contractile recovery by preventing further LVSD decline, recurrent MIs, lethal ventricular arrhythmias, progressive left ventricular (LV) dilation, and sudden cardiac death [24]. For the reasons mentioned above, untreated culprit lesions are responsible for future cardiac events and worse prognosis despite medical treatment. Another critical issue is the timing of revascularization. In patients with NSTEMI and high-risk features such as severe LVSD, early revascularization is associated with an improved LVEF and a favorable prognosis, whereas delayed revascularization in these patients is associated with worse outcomes [6, 25]. Although the clinical outcome, according to the timing of revascularization, was not the main focus of this study, all non-IRA PCIs were performed during the index hospitalization. Therefore, our study provides important insights into the comparative effectiveness of the MV-PCI strategy and the IRA-only PCI strategy in patients with NSTEMI and MVD complicated by severe LVSD, which could help physicians choose the optimal revascularization strategy in clinical practice.

### Strengths and limitations of the study

There are several important implications of this study. First, to the best of our knowledge, this is the first study to evaluate long-term clinical outcomes according to two different revascularization strategies, namely, IRA-only PCI and MV-PCI, in patients with NSTEMI and MVD accompanied by severe LVSD. This study exclusively enrolled patients with NSTEMI presenting with severe LVSD who successfully underwent PCI with newer-generation DESs in tertiary hospitals in Korea, reflecting “real world” clinical practice. Despite the small sample size, the results of this study were based on significant clinical outcomes, such as MACEs, all-cause mortality, and cardiac death. As the current guidelines emphasize the importance of the individualization of interventional strategies based on clinical status, comorbidities, and disease severity in patients with NSTEMI and MVD [12, 13, 18], the present study provides evidence regarding the optimal revascularization strategy in high-risk NSTEMI patients. Second, we found similar in-hospital outcomes and complication rates between the IRA-only PCI and MV PCI groups but better long-term clinical outcomes with the MV-PCI strategy than with the IRA-only PCI strategy, although patients in the study population had a higher risk than those in the previous BCIS study [9]. In the BCIS registry [9], the MV-PCI strategy was associated with an increased risk of in-hospital morbidity and mortality despite being associated with a lower long-term mortality rate than the IRA-only PCI strategy. Additionally, severe LVSD was a strong predictor of mortality, as shown in the subgroup analysis. However, the results should be interpreted with caution as the number of patients with severe LVSD was relatively small (5.6%), and the baseline clinical, lesional, and procedural characteristics of the patients were different from those of our study population (among the patients included in this study, 61.8% had hypertension, 53.1% had diabetes, 53.1% had CKD, and 100% had severe LVSD; in contrast, the study population in the BCIS study [9] was composed of 57.7% hypertensive patients, 34.5% diabetic patients, 6.3% CKD patients, and 5.6% severe LVSD patients). Compared with the BCIS study [9], this study population exclusively included patients who underwent successful PCI (100% vs. 92.1%), had a higher prevalence of DES implantation (96.5% vs. 76.6%), were more likely to receive intravascular ultrasound (IVUS)-guided PCI (21.1% vs. 2.9%) and were more likely to undergo PCI via the transradial approach (64.9% vs. 29.8%). Therefore, the use of these newer-generation DESs, transradial approaches and IVUS-guided PCI possibly resulted in similar in-hospital outcomes between the IRA-only PCI and MV-PCI groups while maintaining the beneficial long-term effect of the MV-PCI strategy. Third, the beneficial effect of the MV-PCI strategy was observed across almost all subgroups of the study population regardless of age, sex, CAD extent, CKD severity, diabetes status, the presence of cardiogenic shock, and the use of mechanical support devices. MV CAD patients with severe LVSD tend to have relatively more comorbidities (e.g., older age, CKD, and diabetes), and more diffuse and complex CAD; however, data comparing different PCI strategies in the various subgroups of these populations are scarce. The exploratory finding in this study might imply that if revascularization was successfully done with the MV-PCI strategy, favorable outcomes might be achieved irrespective of age, patient comorbidities, extent and severity of CAD, and initial clinical presentation.

This study has some limitations. First, our results were derived from a subanalysis of a nationwide, observational, and all-comer AMI registry, including patients with or without cardiogenic shock and a relatively small sample size. These characteristics may have affected the internal and external validity of this study. Additionally, our analysis was underpowered to detect a clinically significant difference in the outcomes; nonsignificant findings may have been significant in a larger cohort of patients. However, we performed several sensitivity analyses to minimize the effect of the possible confounders of different baseline characteristics. A larger clinical study could improve the internal validity of the study. Second, although cardiogenic shock occurred in 14% of the patients in the IRA-only group and 10.5% in the MV-PCI group in the propensity score-matched analysis, which was not a statistically significant difference (and despite the neutral results of the 1-month landmark analysis), this difference in the rate of cardiogenic shock may have impacted the clinical outcomes. In the subgroup analysis, MV-PCI was beneficial in patients with or without cardiogenic shock. Nevertheless, our study results cannot be applied to patients with NSTEMI and cardiogenic shock because only a few cardiogenic shock patients were included. Third, in this study, only 22% of the patients underwent IVUS- or optical coherence tomography (OCT)-guided PCI. Although the routine use of intravascular imaging is not currently recommended, these intracoronary imaging modalities help interventional cardiologists optimize stent implantation, improve clinical outcomes, and reduce periprocedural complications. Fourth, we performed an angiographic evaluation of the lesion severity in the non-IRA. Fractional flow reserve or myocardial perfusion imaging may help assess the nonculprit artery and avoid unnecessary procedures associated with increased in-hospital complications and costs. Fifth, the critical issue of the timing of non-IRA PCI was not addressed in this study due to the small number of MV-PCI patients. However, all non-IRA PCI procedures were done before discharge. Sixth, we could not determine the effect of complete revascularization in this study because only 60.5% of the MV-PCI group achieved complete revascularization. Seventh, in this study, study population performed PCI rather than CABG even though about a half of study patients were younger (43.4% of patients were ≤70 years old), had the three-vessel disease (46.1% of patients), and had diabetes (53.1% of patients), who might be a possible candidate for CABG. In the KAMIR-NIH registry, the choice of revascularization strategy (CABG vs. PCI) was left only to the discretion of the attending physician. Unfortunately, we did not collect any information on the revascularization strategy decision process. Currently, there is no guideline of optimal revascularization strategy (CABG vs. PCI) in patients with NSTEMI and MVD presenting with severe LVSD. Further study will be needed to examine the outcomes depending on revascularization strategy (CABG vs. PCI) in these populations. Eighth, the presence of CTO (chronic total occlusions) in patients with NSTEMI and MVD is known to associate with worse short-term and long-term prognoses [26]. Unfortunately, data on the presence and attempted revascularization of CTO are not available in the KAMIR-NIH registry. However, we only included patients who had successful PCI to minimize the effect of CTO PCI on the present study outcomes. Finally, the KAMIR-NIH registry does not fully capture information about various heart failure (HF) medications (such as aldosterone antagonists, ivabradine, isosorbide dinitrate, hydralazine, and diuretic agents) and HF-related device therapies (such as implantable cardioverter defibrillator, cardiac resynchronization therapy). But as shown in S4 Table, over 90% of our study patients received antiplatelet and statin at discharge. Furthermore, a majority (over 70%) of this study population was taking guideline-directed medical therapy (GDMT) (which includes antiplatelet agents, statin, beta-blocker, ACE inhibitor/ARB) at discharge and successively maintained the usage of GDMT for 3 years following PCI, including statin (93.1%), beta-blockers (78.5%), ACE inhibitor/ARB (67.7%) and aspirin (76.9%), representing optimal GDMT adherence.

## Conclusions

In this prospective, observational, multicenter registry study, including patients with NSTEMI and MVD with severe LVSD, MV-PCI yielded better clinical outcomes than IRA-only PCI with respect to 3-year MACEs. Additionally, the MV-PCI strategy resulted in in-hospital outcomes comparable to those associated with the IRA-only PCI strategy. This study suggests to physicians that the MV-PCI strategy may be beneficial in NSTEMI patients with MVD and severe LVSD. Nevertheless, large-scale, more rigorous observational studies and randomized trials are needed to determine the optimal revascularization strategy in NSTEMI patients without cardiogenic shock.

## Supporting information

S1 Fig Incidence of multivessel disease in patients with NSTEMI following PCI. (TIF)

S1 Table Baseline demographic, clinical and laboratory characteristics in a propensity score-matched population stratified by revascularization strategy and the percent standardized differences in variables among unadjusted, propensity score-matched, and IPW-adjusted populations. (DOCX)

S2 Table Baseline lesional and procedural characteristics in the propensity score-matched population stratified by revascularization strategy and percent standardized differences in variables among unadjusted, propensity score-matched, and IPW-adjusted populations. (DOCX)

S3 Table Independent predictors of MACE at 3 years. (DOCX)

S4 Table Average usage of guideline-directed medical therapy in the study population by period of follow-up. (DOCX)

S1 Dataset A simplified and fully anonymized raw data set. (XLSX)

## Abbreviations

CAD: coronary artery disease
DES: drug-eluting stent(s)
IPTW: inverse probability of treatment weighting
IRA: infarct-related artery
LVEF: left ventricular ejection fraction
MACE: major adverse cardiac events
MI: myocardial infarction
MVD: multivessel disease
NSTEMI: non-ST-segment elevation myocardial infarction
PCI: percutaneous coronary intervention

## References

[1] Khera S, Kolte D, Aronow WS, Palaniswamy C, Subramanian KS, Hashim T, et al. Non-ST-elevation myocardial infarction in the United States: contemporary trends in incidence, utilization of the early invasive strategy, and in-hospital outcomes. J Am Heart Assoc. 2014;3(4):e000995. Epub 2014/07/31. doi: 10.1161/JAHA.114.000995 . 25074695 PMC4310389

[2] Jennings SM, Bennett K, Lonergan M, Shelley E. Trends in hospitalisation for acute myocardial infarction in Ireland, 1997–2008. Heart. 2012;98(17):1285–9. Epub 2012/07/18. doi: 10.1136/heartjnl-2012-301822 . 22802000

[3] Kim Y, Ahn Y, Cho MC, Kim CJ, Kim YJ, Jeong MH. Current status of acute myocardial infarction in Korea. Korean J Intern Med. 2019;34(1):1–10. Epub 2018/12/28.. 30612415 10.3904/kjim.2018.381 PMC6325441

[4] Toma M, Buller CE, Westerhout CM, Fu Y, O’Neill WW, Holmes DR Jr, et al. Non-culprit coronary artery percutaneous coronary intervention during acute ST-segment elevation myocardial infarction: insights from the APEX-AMI trial. European Heart Journal. 2010;31(14):1701–7. doi: 10.1093/eurheartj/ehq129 20530505

[5] Rasoul S, Ottervanger JP, de Boer M-J, Dambrink J-HE, Hoorntje JCA, Marcel Gosselink AT, et al. Predictors of 30-day and 1-year mortality after primary percutaneous coronary intervention for ST-elevation myocardial infarction. Coron Artery Dis. 2009;20(6):415–21. doi: 10.1097/MCA.0b013e32832e5c4c 00019501-200909000-00009. 19641460

[6] Mehta SR, Granger CB, Boden WE, Steg PG, Bassand J-P, Faxon DP, et al. Early versus Delayed Invasive Intervention in Acute Coronary Syndromes. New England Journal of Medicine. 2009;360(21):2165–75. doi: 10.1056/NEJMoa0807986 . 19458363

[7] Ahmed H, Sorin JB, Alexandra JL, Ke X, Gregg WS. Prognostic impact of multivessel versus culprit vessel only percutaneous intervention for patients with multivessel coronary artery disease presenting with acute coronary syndrome. EuroIntervention. 2015;11(3):293–300. doi: 10.4244/EIJY14M08_05 25136882

[8] Emond M, Mock MB, Davis KB, Fisher LD, Holmes DR, Chaitman BR, et al. Long-term survival of medically treated patients in the Coronary Artery Surgery Study (CASS) Registry. Circulation. 1994;90(6):2645–57. doi: 10.1161/01.cir.90.6.2645 7994804

[9] Rathod KS, Koganti S, Jain AK, Astroulakis Z, Lim P, Rakhit R, et al. Complete Versus Culprit-Only Lesion Intervention in Patients With Acute Coronary Syndromes. J Am Coll Cardiol. 2018;72(17):1989–99. Epub 2018/10/20. doi: 10.1016/j.jacc.2018.07.089 . 30336821

[10] Kim MC, Hyun JY, Ahn Y, Bae S, Hyun DY, Cho KH, et al. Optimal Revascularization Strategy in Non-ST-Segment-Elevation Myocardial Infarction With Multivessel Coronary Artery Disease: Culprit-Only Versus One-Stage Versus Multistage Revascularization. J Am Heart Assoc. 2020;9(15):e016575. Epub 2020/08/05. doi: 10.1161/JAHA.120.016575 . 32750302 PMC7792267

[11] Kim MC, Jeong MH, Ahn Y, Kim JH, Chae SC, Kim YJ, et al. What is optimal revascularization strategy in patients with multivessel coronary artery disease in non-ST-elevation myocardial infarction? Multivessel or culprit-only revascularization. International Journal of Cardiology. 2011;153(2):148–53. doi: 10.1016/j.ijcard.2010.08.044 20843572

[12] Collet J-P, Thiele H, Barbato E, Barthélémy O, Bauersachs J, Bhatt DL, et al. 2020 ESC Guidelines for the management of acute coronary syndromes in patients presenting without persistent ST-segment elevation: The Task Force for the management of acute coronary syndromes in patients presenting without persistent ST-segment elevation of the European Society of Cardiology (ESC). European Heart Journal. 2020. doi: 10.1093/eurheartj/ehaa575 32860058

[13] Amsterdam EA, Wenger NK, Brindis RG, Casey DE, Ganiats TG, Holmes DR, et al. 2014 AHA/ACC Guideline for the Management of Patients With Non -ST-Elevation Acute Coronary Syndromes: A Report of the American College of Cardiology/American Heart Association Task Force on Practice Guidelines. J Am Coll Cardiol. 2014;64(24):e139–e228. doi: 10.1016/j.jacc.2014.09.017 25260718

[14] Kim JH, Chae S-C, Oh DJ, Kim H-S, Kim YJ, Ahn Y, et al. Multicenter Cohort Study of Acute Myocardial Infarction in Korea Interim Analysis of the Korea Acute Myocardial Infarction Registry National Institutes of Health Registry. Circ J. 2016;80(6):1427–36. doi: 10.1253/circj.CJ-16-0061 27118621

[15] Thygesen K, Alpert JS, Jaffe AS, Chaitman BR, Bax JJ, Morrow DA, et al. Fourth Universal Definition of Myocardial Infarction (2018). J Am Coll Cardiol. 2018;72(18):2231–64. Epub 2018/08/30. doi: 10.1016/j.jacc.2018.08.1038 . 30153967

[16] Velazquez EJ, Lee KL, Deja MA, Jain A, Sopko G, Marchenko A, et al. Coronary-Artery Bypass Surgery in Patients with Left Ventricular Dysfunction. New England Journal of Medicine. 2011;364(17):1607–16. doi: 10.1056/NEJMoa1100356 . 21463150 PMC3415273

[17] Yancy CW, Jessup M, Bozkurt B, Butler J, Casey DE, Drazner MH, et al. 2013 ACCF/AHA Guideline for the Management of Heart Failure. Circulation. 2013;128(16):e240–e327. doi: 10.1161/CIR.0b013e31829e8776 23741058

[18] Neumann F-J, Sousa-Uva M, Ahlsson A, Alfonso F, Banning AP, Benedetto U, et al. 2018 ESC/EACTS Guidelines on myocardial revascularization. European Heart Journal. 2018;40(2):87–165. doi: 10.1093/eurheartj/ehy394 30165437

[19] Roffi M, Patrono C, Collet JP, Mueller C, Valgimigli M, Andreotti F, et al. 2015 ESC Guidelines for the management of acute coronary syndromes in patients presenting without persistent ST-segment elevation: Task Force for the Management of Acute Coronary Syndromes in Patients Presenting without Persistent ST-Segment Elevation of the European Society of Cardiology (ESC). Eur Heart J. 2016;37(3):267–315. Epub 2015/09/01. doi: 10.1093/eurheartj/ehv320 . 26320110

[20] Kastrati A, Schömig A, Elezi S, Dirschinger J, Mehilli J, Schühlen H, et al. Prognostic value of the modified american college of Cardiology/American heart association stenosis morphology classification for long-term angiographic and clinical outcome after coronary stent placement. Circulation. 1999;100(12):1285–90. Epub 1999/09/24. doi: 10.1161/01.cir.100.12.1285 . 10491372

[21] Niccoli G, Scalone G, Crea F. Acute myocardial infarction with no obstructive coronary atherosclerosis: mechanisms and management. European Heart Journal. 2014;36(8):475–81. doi: 10.1093/eurheartj/ehu469 25526726

[22] Panza JA, Velazquez EJ, She L, Smith PK, Nicolau JC, Favaloro RR, et al. Extent of Coronary and Myocardial Disease and Benefit From Surgical Revascularization in LV Dysfunction. Journal of the American College of Cardiology. 2014;64(6):553–61. doi: 10.1016/j.jacc.2014.04.064 25104523 PMC4129547

[23] Bonow RO, Maurer G, Lee KL, Holly TA, Binkley PF, Desvigne-Nickens P, et al. Myocardial Viability and Survival in Ischemic Left Ventricular Dysfunction. New England Journal of Medicine. 2011;364(17):1617–25. doi: 10.1056/NEJMoa1100358 . 21463153 PMC3290901

[24] Samady H, Elefteriades JA, Abbott BG, Mattera JA, McPherson CA, Wackers FJT. Failure to Improve Left Ventricular Function After Coronary Revascularization for Ischemic Cardiomyopathy Is Not Associated With Worse Outcome. Circulation. 1999;100(12):1298–304. doi: 10.1161/01.cir.100.12.1298 10491374

[25] Bax JJ, Schinkel AFL, Boersma E, Rizzello V, Elhendy A, Maat A, et al. Early Versus Delayed Revascularization in Patients With Ischemic Cardiomyopathy and Substantial Viability: Impact on Outcome. Circulation. 2003;108(10_suppl_1):II-39–II-42. doi: 10.1161/01.cir.0000089041.69175.9d 12970206

[26] Gierlotka M, Tajstra M, Gąsior M, Hawranek M, Osadnik T, Wilczek K, et al. Impact of chronic total occlusion artery on 12-month mortality in patients with non-ST-segment elevation myocardial infarction treated by percutaneous coronary intervention (from the PL-ACS Registry). Int J Cardiol. 2013;168(1):250–4. Epub 2012/10/13. doi: 10.1016/j.ijcard.2012.09.086 . 23058348

