# Supplementary figures and images for: Multivessel versus IRA-only PCI in patients with NSTEMI and severe left ventricular systolic dysfunction
Source: PLoS One. 2021 Oct 13;16(10):e0258525. doi: 10.1371/journal.pone.0258525 (PMC8513855; doi:10.1371/journal.pone.0258525)

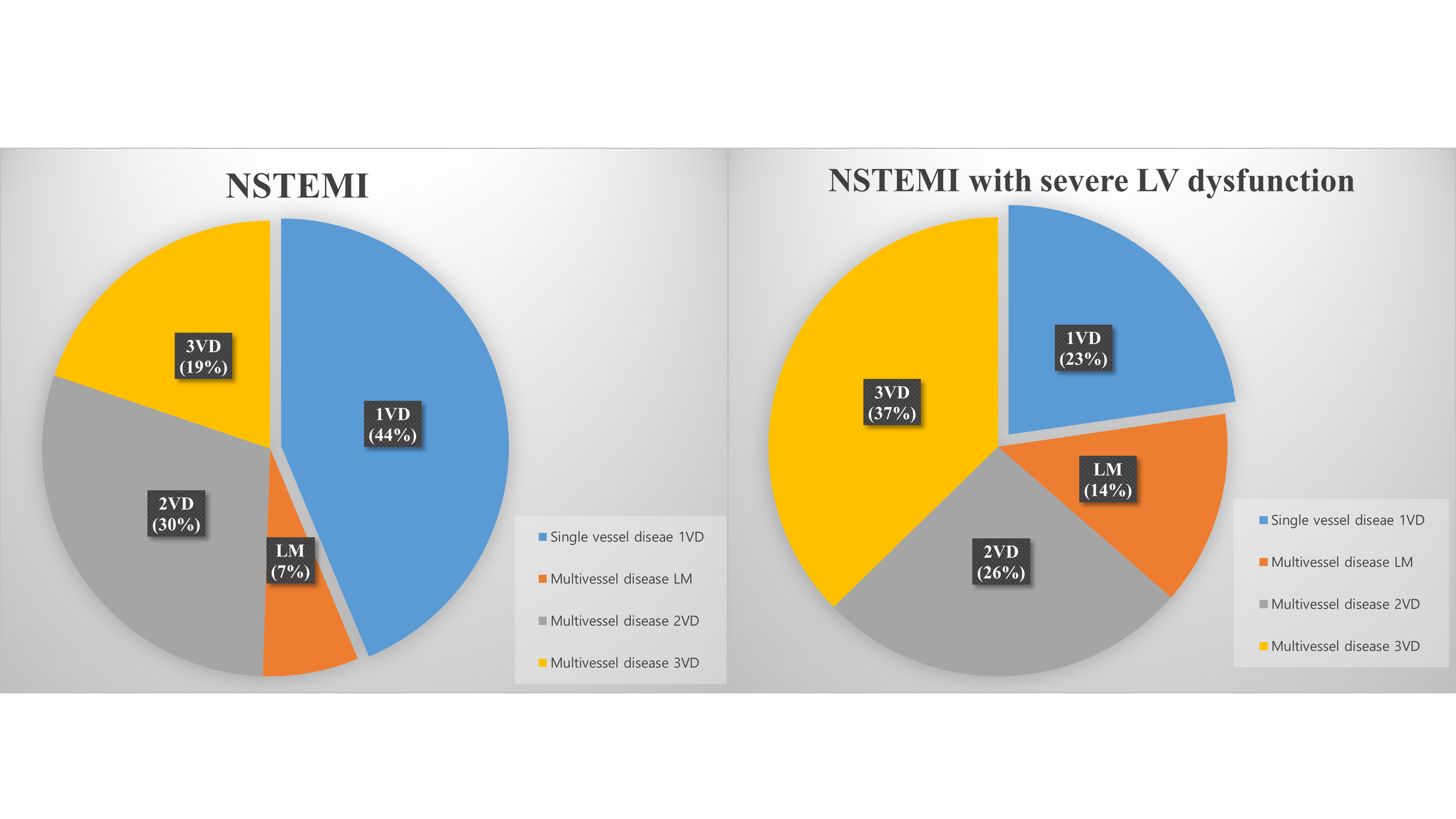

Supplement: S1 Fig — (TIF) [file pone.0258525.s001.tif]
