# Supplementary material for: Multivessel versus IRA-only PCI in patients with NSTEMI and severe left ventricular systolic dysfunction
Source: PLoS One. 2021 Oct 13;16(10):e0258525. doi: 10.1371/journal.pone.0258525 (PMC8513855; doi:10.1371/journal.pone.0258525)
Supplement: S1 Table — (DOCX) [file pone.0258525.s002.docx]

**S1 Table. Baseline demographic, clinical and laboratory characteristics in a propensity score-matched population stratified by revascularization strategy and the percent standardized differences in variables among unadjusted, propensity score-matched, and IPW-adjusted populations**

|  |  |  | |  |  | |  | |  |
| --- | --- | --- | --- | --- | --- | --- | --- | --- | --- |
| **Characteristic** | **Total population** | **IRA-Only PCI** | **Multivessel PCI** | **p-value** | **Standardized**  **difference** | | | |  |
|  | **(n=172)** | **(n=86)** | **(n=86)** |  | **Unadjusted** | **PS-matched** | | **IPW-adjusted** |  |
| **Demographic** |  |  |  |  |  |  | |  |  |
| **Age (years)** | 70.5±10.4 | 70.6±11.2 | 70.3±9.6 | 0.780 | -0.268 | -0.035 | | -0.002 |  |
| **Age > 70 years** | 103(59.9) | 53(61.6) | 50(58.1) | 0.736 | -0.221 | -0.071 | | 0.008 |  |
| **Male** | 117(68.0) | 60(69.8) | 57(66.3) | 0.743 | -0.109 | -0.075 | | 0.010 |  |
| **BMI (kg/m^2^)** | 22.5±3.5 | 22.7±3.9 | 22.3±3.0 | 0.445 | -0.045 | -0.116 | | -0.030 |  |
| **Initial presentation** |  |  |  |  |  |  | |  |  |
| **SBP (mmHg)** | 126.8±33.7 | 125.9±35.7 | 127.7±31.7 | 0.722 | 0.006 | 0.054 | | 0.095 |  |
| **DBP (mmHg)** | 76.4±20.1 | 75.9±21.5 | 77.0±18.7 | 0.741 | 0.019 | 0.054 | | 0.110 |  |
| **Heart rate (frequency/min)** | 95.2±23.9 | 94.3±24.1 | 96.2±23.8 | 0.574 | -0.044 | 0.081 | | -0.025 |  |
| **Killip classification (%)** | 75(43.6) | 36(41.9) | 39(45.4) | 0.761 | -0.045 | -0.070 | | -0.005 |  |
| III, IV |  |  |  |  |  |  | |  |  |
| **Symptom onset-to-arrival time, days** | 1.8±3.3 | 1.3±1.8 | 2.2±4.3 | 0.064 | 0.191 | 0.286 | | 0.150 |  |
| **Arrival-to-angiography time, days** | 2.3±5.2 | 2.7±6.7 | 1.9±2.9 | 0.321 | -0.144 | -0.154 | | -0.140 |  |
| **Clinical risk factors** |  |  |  |  |  |  | |  |  |
| **Diabetes (%)** | 97(56.4) | 46(53.5) | 51(59.3) | 0.542 | -0.064 | 0.117 | | -0.075 |  |
| **Hypertension (%)** | 104(60.5) | 48(55.8) | 56(65.1) | 0.291 | 0.084 | 0.191 | | 0.225 |  |
| **Dyslipidemia (%)** | 19(11.0) | 7(8.1) | 12(14.0) | 0.359 | 0.123 | 0.186 | | 0.231 |  |
| **Previous MI (%)** | 25(14.5) | 13(15.1) | 12(14.0) | >0.999 | -0.071 | -0.033 | | -0.023 |  |
| **Prior CVA (%)** | 25(14.5) | 15(17.4) | 10(11.6) | 0.424 | -0.071 | -0.166 | | -0.075 |  |
| **CKD (%)** | 95(55.2) | 43(50.0) | 52(60.5) | 0.222 | 0.042 | 0.212 | | 0.098 |  |
| **Current smoking (%)** | 39(22.7) | 21(24.4) | 18(20.9) | 0.711 | 0.236 | -0.083 | | 0.040 |  |
| **Laboratory findings** |  |  |  |  |  |  | |  |  |
| **Hb (g/dL)** | 12.4±2.3 | 12.5±2.3 | 12.2±2.2 | 0.415 | 0.124 | -0.116 | | 0.031 |  |
| **Creatinine (mg/dL)** | 1.9±2.0 | 1.7±1.6 | 2.2±2.3 | 0.140 | 0.070 | 0.229 | | 0.076 |  |
| **eGFR (mL/min/1.72m^2^)** | 58.6±34.0 | 61.5±33.7 | 55.7±34.1 | 0.294 | 0.039 | -0.171 | | -0.049 |  |
| **HbA1c (%)** | 7.0±1.7 | 6.8±1.6 | 7.1±1.8 | 0.234 | 0.197 | 0.183 | | 0.098 |  |
| **CK-MB (ng/mL)** | 70.0±114.2 | 71.6±126.8 | 68.5±100.7 | 0.864 | 0.043 | -0.027 | | -0.017 |  |
| **Troponin I (ng/mL)** | 39.5±80.3 | 37.2±67.4 | 41.9±91.7 | 0.676 | 0.197 | 0.058 | | 0.073 |  |
| **LDL cholesterol (mg/dL)** | 98.8±36.8 | 98.2±36.6 | 99.4±37.2 | 0.815 | 0.305 | 0.032 | | 0.034 |  |
| **HDL cholesterol (mg/dL)** | 42.0±12.4 | 43.1±13.0 | 41.0±11.6 | 0.210 | -0.189 | -0.173 | | -0.165 |  |
| **hsCRP (mg/L)** | 3.9±4.1 | 3.3±3.6 | 4.5±4.4 | 0.076 | 0.076 | 0.283 | | 0.104 |  |
| **NT-proBNP (pg/mL)** | 10,663.6±10,124.7 | 9,183.3±9,751.3 | 12,143.9±10,329.8 | 0.071 | -0.031 | 0.295 | | 0.129 |  |
| **LVEF (%)** | 27.8±5.1 | 27.7±4.8 | 27.9±5.4 | 0.827 | 0.031 | 0.032 | | 0.035 |  |
| **Medications at discharge** |  |  |  |  |  |  | |  |  |
| **Aspirin (%)** | 165(95.9) | 82(95.4) | 83(96.5) | >0.999 | 0.082 | 0.059 | | 0.110 |  |
| **Clopidogrel (%)** | 148(86.0) | 73(84.9) | 75(87.2) | 0.815 | -0.343 | 0.067 | | 0.021 |  |
| **Prasugrel (%)** | 9(5.2) | 3(3.5) | 6(7.0) | 0.508 | 0.283 | 0.157 | | 0.068 |  |
| **Ticagrelor (%)** | 9(5.2) | 7(8.1) | 2(2.3) | 0.180 | 0.209 | -0.263 | | -0.063 |  |
| **Potent P2Y12 inhibitors (%)** | 18(10.5) | 10(11.6) | 8(9.3) | 0.774 | 0.358 | -0.076 | | -0.009 |  |
| **ACE inhibitor/ARB (%)** | 127(73.8) | 61(70.9) | 66(76.7) | 0.424 | 0.103 | 0.133 | | 0.201 |  |
| **ß-blocker (%)** | 139(80.8) | 66(76.7) | 73(84.9) | 0.230 | 0.157 | 0.208 | | 0.202 |  |
| **Statin (%)** | 141(82.0) | 70(81.4) | 71(82.6) | >0.999 | 0.173 | 0.030 | | 0.204 |  |
| **Oral anticoagulant (%)** | 17(9.9) | 8(9.3) | 9(10.5) | >0.999 | 0.062 | 0.039 | | 0.055 |  |
|  |  |  |  |  |  | |  | |  |
|  |  |  |  |  |  | |  | |  |
|  |  |  |  |  |  | |  | |  |

Data are presented as mean ± SD, median (interquartile range), and number (percentage) as appropriate.

Abbreviations: IRA, infarct-related artery; BMI, body mass index; SBP, systolic blood pressure; DBP, diastolic blood pressure; MI, myocardial infarction; CVA, cerebrovascular accident; CKD, chronic kidney disease; Hb, hemoglobin; eGFR, estimated glomerular filtration rate; HbA1c, glycated hemoglobin A1c; LDL, low-density lipoprotein; HDL, high-density lipoprotein; hsCRP, high-sensitivity c-reactive protein; NT-proBNP, N-terminal prohormone of brain natriuretic peptide; LVEF, left ventricular ejection fraction; ACE inhibitor, angiotensin-converting enzyme inhibitor; ARB, angiotensin receptor blocker.
