# Supplementary material for: Multivessel versus IRA-only PCI in patients with NSTEMI and severe left ventricular systolic dysfunction
Source: PLoS One. 2021 Oct 13;16(10):e0258525. doi: 10.1371/journal.pone.0258525 (PMC8513855; doi:10.1371/journal.pone.0258525)
Supplement: S2 Table — (DOCX) [file pone.0258525.s003.docx]

| **S2 Table. Baseline lesional and procedural characteristics in the propensity score-matched population stratified by revascularization strategy and percent standardized differences in variables among unadjusted, propensity score-matched, and IPW-adjusted populations**   \|  \|  \|  \| \|  \|  \| \| \| \|  \| \| \| \| --- \| --- \| --- \| --- \| --- \| --- \| --- \| --- \| --- \| --- \| --- \| --- \| \| **Characteristic** \| **Total population** \| **IRA-Only PCI** \| **Multivessel PCI** \| **p-value** \| **Standardized**  **difference** \| \| \| \| \| \| \| \| \| **(n=172)** \| **(n=86)** \| **(n=86)** \| **Unadjusted** \| \| **PS-matched** \| \| \| **IPW-adjusted** \| \| \| \| \| **Culprit vessel (%)** \|  \|  \|  \| 0.269 \| \| 0.294 \| \| 0.276 \| \| \| 0.287 \| \| \| \| LAD \| 69(40.1) \| 40(46.5) \| 29(33.7) \|  \| \|  \| \|  \| \| \|  \| \| \| \| LCX \| 33(19.2) \| 15(17.4) \| 18(20.9) \|  \| \|  \| \|  \| \| \|  \| \| \| \| RCA \| 54(31.4) \| 23(26.7) \| 31(36.1) \|  \| \|  \| \|  \| \| \|  \| \| \| \| LMCA \| 16(9.3) \| 8(9.3) \| 8(9.3) \|  \| \|  \| \|  \| \| \|  \| \| \| \| **Lesion classification (%)** \|  \|  \|  \|  \| \|  \| \|  \| \| \|  \| \| \| \| **B2/C** \| 157(91.3) \| 78(90.7) \| 79(91.9) \| >0.999 \| \| -0.117 \| \| -0.041 \| \| \| -0.068 \| \| \| \| **Small vessel** \| 89(51.7) \| 40(46.5) \| 49(57.0) \| 0.253 \| \| 0.113 \| \| 0.211 \| \| \| 0.155 \| \| \| \| **Long lesion** \| 92(53.5) \| 40(46.5) \| 52(60.5) \| 0.111 \| \| 0.207 \| \| 0.283 \| \| \| 0.204 \| \| \| \| **Overall lesion profile (%)** \|  \|  \|  \|  \| \|  \| \|  \| \| \|  \| \| \| \| **Left Main disease** \| 27(15.7) \| 13(15.1) \| 14(16.3) \| >0.999 \| \| 0.267 \| \| 0.032 \| \| \| -0.032 \| \| \| \| **Three-vessel disease** \| 84(48.8) \| 41(47.7) \| 43(50.0) \| 0.860 \| \| -0.075 \| \| 0.047 \| \| \| 0.085 \| \| \| \| **Pre TIMI flow of culprit vessel (%)** \|  \|  \|  \| 0.203 \| \| 0.200 \| \| 0.162 \| \| \| 0.205 \| \| \| \| 0 \| 43(25.0) \| 19(22.1) \| 24(27.9) \|  \| \|  \| \|  \| \| \|  \| \| \| \| I \| 30(17.4) \| 17(19.8) \| 13(15.1) \|  \| \|  \| \|  \| \| \|  \| \| \| \| II, III \| 99(57.6) \| 50(58.1) \| 49(57.0) \|  \| \|  \| \|  \| \| \|  \| \| \| \| **Post TIMI flow of culprit vessel (%)** \|  \|  \|  \|  \| \|  \| \|  \| \| \|  \| \| \| \| II, III \| 172(100.0) \| 86(100.0) \| 86(100.0) \|  \| \|  \| \|  \| \| \|  \| \| \| \| **IVUS during PCI (%)** \| 32(18.6) \| 15(17.4) \| 17(19.8) \| 0.824 \| \| 0.215 \| \| 0.060 \| \| \| 0.094 \| \| \| \| **OCT during PCI (%)** \| 2(1.2) \| 2(2.3) \| 0(0.0) \| 0.500 \| \| -0.198 \| \| -0.218 \| \| \| -0.207 \| \| \| \| **IRA treatment (%)** \|  \|  \|  \| 0.515 \| \| 0.239 \| \| 0.237 \| \| \| 0.215 \| \| \| \| Bare-metal stent \| 7(4.1) \| 5(5.8) \| 2(2.3) \|  \| \|  \| \|  \| \| \|  \| \| \| \| First-generation DES \| 1(0.6) \| 1(1.2) \| 0(0.0) \|  \| \|  \| \|  \| \| \|  \| \| \| \| Second-generation DES \| 164(95.3) \| 80(93.0) \| 84(97.7) \|  \| \|  \| \|  \| \| \|  \| \| \| \| **Total number of implanted stents** \| 1.9±1.0 \| 1.3±0.6 \| 2.6±0.9 \|  \| \|  \| \| 1.573 \| \| \|  \| \| \| \| **Timing of non-IRA PCI** \|  \|  \|  \| <0.001 \| \| -0.976 \| \| -0.957 \| \| \| -0.976 \| \| \| \| Single-staged PCI during index procedure \| 145(84.3) \| 86(100.0) \| 59(68.6) \|  \| \|  \| \|  \| \| \|  \| \| \| \| Multi-staged PCI during index hospitalization \| 27(15.7) \| 0(0.0) \| 27(31.4) \|  \| \|  \| \|  \| \| \|  \| \| \| \| **Complete revascularization** \| - \| - \| 51(59.3) \| - \| \| - \| \| - \| \| \| - \| \| \| \| **Hemodynamic support** \|  \|  \|  \|  \| \|  \| \|  \| \| \|  \| \| \| \| Intra-aortic balloon pump \| 11(6.4) \| 4(4.7) \| 7(8.1) \| 0.549 \| \| 0.133 \| \| 0.143 \| \| \| 0.089 \| \| \| \| Extracorporeal membrane oxygenation \| 4(2.3) \| 3(3.5) \| 1(1.2) \| 0.625 \| \| -0.137 \| \| -0.155 \| \| \| -0.183 \| \| \| \| **Approach site** \|  \|  \|  \|  \| \|  \| \|  \| \| \|  \| \| \| \| Transfemoral approach \| 58(33.7) \| 28(32.6) \| 30(34.9) \| 0.871 \| \| -0.018 \| \| -0.049 \| \| \| -0.054 \| \| \| \| **Glycoprotein IIb/IIIa inhibitor** \| 19(11.0) \| 12(14.0) \| 7(8.1) \| 0.359 \| \| -0.176 \| \| -0.186 \| \| \| -0.270 \| \| \| \| **Thrombus aspiration** \| 17(9.9) \| 10(11.6) \| 7(8.1) \| 0.581 \| \| -0.055 \| \| -0.117 \| \| \| -0.110 \| \| \| |
| --- | --- | --- | --- | --- | --- | --- | --- | --- | --- | --- | --- | --- | --- | --- | --- | --- | --- | --- | --- | --- | --- | --- | --- | --- | --- | --- | --- | --- | --- | --- | --- | --- | --- | --- | --- | --- | --- | --- | --- | --- | --- | --- | --- | --- | --- | --- | --- | --- | --- | --- | --- | --- | --- | --- | --- | --- | --- | --- | --- | --- | --- | --- | --- | --- | --- | --- | --- | --- | --- | --- | --- | --- | --- | --- | --- | --- | --- | --- | --- | --- | --- | --- | --- | --- | --- | --- | --- | --- | --- | --- | --- | --- | --- | --- | --- | --- | --- | --- | --- | --- | --- | --- | --- | --- | --- | --- | --- | --- | --- | --- | --- | --- | --- | --- | --- | --- | --- | --- | --- | --- | --- | --- | --- | --- | --- | --- | --- | --- | --- | --- | --- | --- | --- | --- | --- | --- | --- | --- | --- | --- | --- | --- | --- | --- | --- | --- | --- | --- | --- | --- | --- | --- | --- | --- | --- | --- | --- | --- | --- | --- | --- | --- | --- | --- | --- | --- | --- | --- | --- | --- | --- | --- | --- | --- | --- | --- | --- | --- | --- | --- | --- | --- | --- | --- | --- | --- | --- | --- | --- | --- | --- | --- | --- | --- | --- | --- | --- | --- | --- | --- | --- | --- | --- | --- | --- | --- | --- | --- | --- | --- | --- | --- | --- | --- | --- | --- | --- | --- | --- | --- | --- | --- | --- | --- | --- | --- | --- | --- | --- | --- | --- | --- | --- | --- | --- | --- | --- | --- | --- | --- | --- | --- | --- | --- | --- | --- | --- | --- | --- | --- | --- | --- | --- | --- | --- | --- | --- | --- | --- | --- | --- | --- | --- | --- | --- | --- | --- | --- | --- | --- | --- | --- | --- | --- | --- | --- | --- | --- | --- | --- | --- | --- | --- | --- | --- | --- | --- | --- | --- | --- | --- | --- | --- | --- | --- | --- | --- | --- | --- | --- | --- | --- | --- | --- | --- | --- | --- | --- | --- | --- | --- | --- | --- | --- | --- | --- | --- | --- | --- | --- | --- | --- | --- | --- | --- | --- | --- | --- | --- | --- | --- | --- | --- | --- | --- | --- | --- | --- | --- | --- | --- | --- | --- | --- | --- | --- | --- | --- | --- | --- | --- | --- | --- | --- | --- | --- | --- | --- | --- | --- | --- | --- | --- | --- | --- | --- | --- | --- | --- | --- | --- | --- | --- | --- | --- | --- | --- | --- | --- | --- | --- | --- | --- | --- | --- | --- | --- | --- | --- | --- | --- | --- | --- | --- | --- | --- | --- | --- | --- | --- | --- | --- | --- | --- | --- | --- | --- | --- | --- | --- | --- | --- | --- | --- | --- | --- | --- | --- | --- | --- | --- | --- | --- | --- | --- | --- | --- | --- | --- | --- | --- | --- | --- | --- | --- | --- | --- | --- | --- | --- | --- | --- | --- | --- | --- | --- | --- | --- | --- | --- | --- | --- | --- | --- | --- | --- | --- | --- | --- | --- | --- | --- | --- | --- | --- | --- | --- | --- | --- | --- | --- | --- | --- | --- | --- | --- | --- | --- | --- | --- | --- | --- | --- | --- | --- | --- | --- | --- | --- | --- | --- | --- | --- | --- | --- | --- | --- | --- | --- | --- | --- | --- | --- | --- | --- | --- | --- | --- | --- | --- | --- | --- | --- | --- | --- | --- | --- | --- | --- | --- | --- | --- | --- | --- | --- | --- | --- | --- | --- | --- | --- | --- | --- | --- | --- | --- | --- | --- | --- | --- | --- |
| Data are presented as mean ± SD, median (interquartile range), and number (percentage) as appropriate.  Abbreviations: LAD, left anterior descending artery; LCX, left circumflex artery; RCA, right coronary artery; LMCA, left main coronary artery, lesion based on American College of Cardiology/American Heart Association lesion classification; TIMI, thrombolysis in myocardial infarction; IVUS, intravascular ultrasound; OCT, optical coherence tomography; DES, drug eluting stent; PCI, percutaneous coronary intervention; GpIIb-IIIa inhibitor, glycoprotein IIb/IIIa inhibitor. |
