# Supplementary material for: Multivessel versus IRA-only PCI in patients with NSTEMI and severe left ventricular systolic dysfunction
Source: PLoS One. 2021 Oct 13;16(10):e0258525. doi: 10.1371/journal.pone.0258525 (PMC8513855; doi:10.1371/journal.pone.0258525)
Supplement: S3 Table — (DOCX) [file pone.0258525.s004.docx]

**S3 Table. Independent predictors of MACE at 3 years**

|  |  |  | | |
| --- | --- | --- | --- | --- |
|  | **Comparator** | | **aHR** | **P value** |
|  |  |  | **(95% CI)** |  |
| Multivessel PCI | IRA-Only PCI | | 0.58 (0.34-0.99) | 0.046 |
| Statin use | No statin use | | 0.36 (0.18-0.70) | 0.003 |
| Troponin I |  | | 1.01 (1.00-1.01) | 0.032 |
|  |  | |  |  |

Adjusted for age, sex, CKD (eGFR < 60 ml/min/1.73 m^2^), aspirin use, beta blocker use, renin angiotensin aldosterone inhibitor use, ECMO use, stent type, timing of non-IRA PCI, Hb, Cr, CK-MB, HbA1c, and NT-proBNP.

Abbreviations: aHR, adjusted hazard ratio; CI, confidence interval; MACE, major adverse cardiac event; PCI, percutaneous coronary intervention.
