# Supplementary material for: Multivessel versus IRA-only PCI in patients with NSTEMI and severe left ventricular systolic dysfunction
Source: PLoS One. 2021 Oct 13;16(10):e0258525. doi: 10.1371/journal.pone.0258525 (PMC8513855; doi:10.1371/journal.pone.0258525)
Supplement: S4 Table — (DOCX) [file pone.0258525.s005.docx]

S4 Table. Average usage of guideline-directed medical therapy in the study population by period of follow-up

|  | **Total (n= 228)** | **IRA-Only PCI(n=104)** | **Multivessel PCI(n=124)** | **p-value** |  |  |  |
| --- | --- | --- | --- | --- | --- | --- | --- |
| **Aspirin** |  |  |  |  |  |  |  |
| Discharge | 221(96.9) | 100(96.2) | 121(97.6) | 0.705 |  |  |  |
| 12 months | 160/174(92.0) | 67/75(89.3) | 93/99(93.9) | 0.269 |  |  |  |
| 24 months | 132/155(85.2) | 49/60(81.7) | 83/95(87.4) | 0.331 |  |  |  |
| 36 months | 100/130(76.9) | 37/48(77.1) | 63/82(76.8) | 0.974 |  |  |  |
| **P2Y12 inhibitor** |  |  |  |  |  |  |  |
| Discharge | 228/228(96.9) | 101/104(97.1) | 120/124(96.8) | 0.882 |  |  |  |
| 12 months | 142/174(81.6) | 60/75(80.0) | 82/99(82.8) | 0.633 |  |  |  |
| 24 months | 114/155(73.5) | 42/60(70.0) | 72/95(75.8) | 0.426 |  |  |  |
| 36 months | 94/130(72.3) | 34/48(70.8) | 60/82(73.2) | 0.774 |  |  |  |
| **Statin** |  |  |  |  |  |  |  |
| Discharge | 190(83.3) | 83(79.8) | 107(86.3) | 0.191 |  |  |  |
| 12 months | 158/174(90.8) | 67/75(89.3) | 91/99(91.9) | 0.559 |  |  |  |
| 24 months | 142/155(91.6) | 53/60(88.3) | 89/95(93.7) | 0.242 |  |  |  |
| 36 months | 121/130(93.1) | 43/48(89.6) | 78/82(95.1) | 0.289 |  |  |  |
| **Beta blocker** |  |  |  |  |  |  |  |
| Discharge | 179(78.5) | 78(75.0) | 101(81.5) | 0.238 |  |  |  |
| 12 months | 142/174(81.6) | 60/75(80.0) | 82/99(82.8) | 0.633 |  |  |  |
| 24 months | 122/155(78.7) | 41/60(68.3) | 81/95(85.3) | 0.012 |  |  |  |
| 36 months | 102/130(78.5) | 32/48(66.7) | 70/82(85.4) | 0.012 |  |  |  |
| **ACE inhibitor/ARB** |  |  |  |  |  |  |  |
| Discharge | 170(74.6) | 75(72.1) | 95(76.6) | 0.437 |  |  |  |
| 12 months | 129/174(74.1) | 52/75(69.3) | 77/99(77.8) | 0.208 |  |  |  |
| 24 months | 101/155(65.2) | 37/60(61.7) | 64/95(67.4) | 0.468 |  |  |  |
| 36 months | 88/130(67.7) | 29/48(60.4) | 59/82(72.0) | 0.175 |  |  |  |
|  |  |  |  |  | |  |  |

Values are mean ± SD, n (%), or median (interquartile range). Bold indicates statistical significance.

Abbreviations: ACE inhibitor, angiotensin-converting enzyme inhibitor; ARB, angiotensin receptor blocker.
